# To Tie or Not to Tie? That Is the Question

**DOI:** 10.3390/polym9090454

**Published:** 2017-09-16

**Authors:** Pawel Dabrowski-Tumanski, Joanna I. Sulkowska

**Affiliations:** 1 Centre of New Technologies, University of Warsaw, Warsaw 02-097, Poland; p.dabrowski@cent.uw.edu.pl; 2 Faculty of Chemistry, University of Warsaw, Warsaw 02-093, Poland

**Keywords:** knots, link, lasso, topology, polymer, disulfide

## Abstract

In this review, we provide an overview of entangled proteins. Around 6% of protein structures deposited in the PBD are entangled, forming knots, slipknots, lassos and links. We present theoretical methods and tools that enabled discovering and classifying such structures. We discuss the advantages and disadvantages of the non-trivial topology in proteins, based on available data about folding, stability, biological properties and evolutionary conservation. We also formulate intriguing and challenging questions on the border of biophysics, bioinformatics, biology and mathematics, which arise from the discovery of an entanglement in proteins. Finally, we discuss possible applications of entangled proteins in medicine and nanotechnology, such as the chance to design super stable proteins, whose stability could be controlled by chemical potential.

## 1. Introduction

**Figure 1:**
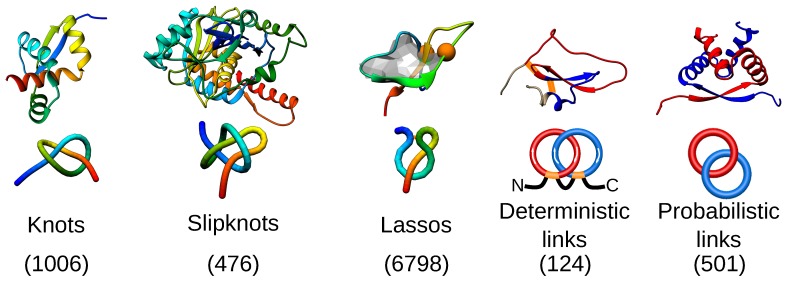
Types of entanglement identified up to now in proteins, represented by the simplest topological motif. From left to right: the trefoil knot (PDB ID 1j85), the trefoil slipknot (PDB ID 1hyn), the single lasso (a covalent loop is pierced once by a tail; PDB ID 1ews), the deterministic (PDB ID 2lfk) and probabilistic (PDB ID 1arr) Hopf link. For each topological group, the exemplary protein structure along with a scheme of the topology is presented. The numbers present the current number of identified structures with a given topological motif based on the databases: KnotProt, LassoProt and LinkProt.

Self-tying of proteins was long considered as impossible, as it requires the threading of a chain through a twisted loop, which in principle is an entropically unfavorable event. However, similar structures have been known to exist in other biopolymers (such as DNA) for many years [1,2], and their self-tying was attributed to, e.g., better packing [3]. Moreover, specialized enzymes dealing with topologically-complex biopolymers have been identified and studied extensively [4,5,6]. In the case of proteins, the situation began to change approximately 20 years ago, by the works of, e.g., Mansfield, Taylor, Nureki and others [7,8,9,10,11,12,13]. Nowadays, every week, new structures are deposited in PDB, resulting in many deeply-knotted and slipknotted proteins (Figure 1) being discovered [14,15].

In the last few years, new types of entanglement in proteins have been discovered: complex lassos and links. The complex lasso is formed based on a covalent loop closed by, e.g., a disulfide bridge, which is pierced by at least one tail [16] (Figure 1). Then, two lasso covalent loops can thread one another, giving rise to deterministic links (Figure 1) [17]. Finally, in a multi-chain protein complex, the chains may wind one around the other, forming a probabilistic link [18]. These entangled proteins have been clustered according to their topology, biological function and structural similarity and are available for further analysis in the databases: KnotProt [14] collecting knots and slipknots, LassoProt [19] for lassos and LinkProt [18], designed for links.

Despite thermodynamical burden introduced by the reduction of entropy by topological constraints, careful search through the entire PDB shows that up to 6% of proteins have a complex topology. Is it much? This is for sure much more than would be predicted by protein researchers even a few years ago. Therefore, it is time to ask: is the entropy reduction coming from threading really so unfavorable for proteins as we once thought, or is this disfavor possibly balanced by threading-induced advantages?

The aim of this review is to present the advantages and disadvantages of the complex topology based on available data and to suggest a possible answer to the question why entangled proteins exist. In the first section, we give a short review of known protein topologies. Next, we summarize available information about the influence of topology on the biological function and biophysics of proteins, analyzing every aspect in detail in the following subsections. Armed with all of the arguments, we return the discussion to the (highly non-trivial) question: are 6% of structures much? Our aim is to deliver the most complete set of references; however, as the topic of complex topology proteins has become a separate, dynamically changing field in protein biophysics, probably, we were not able to include every relevant work.

## 2. Entanglement in Proteins

**Figure 2:**
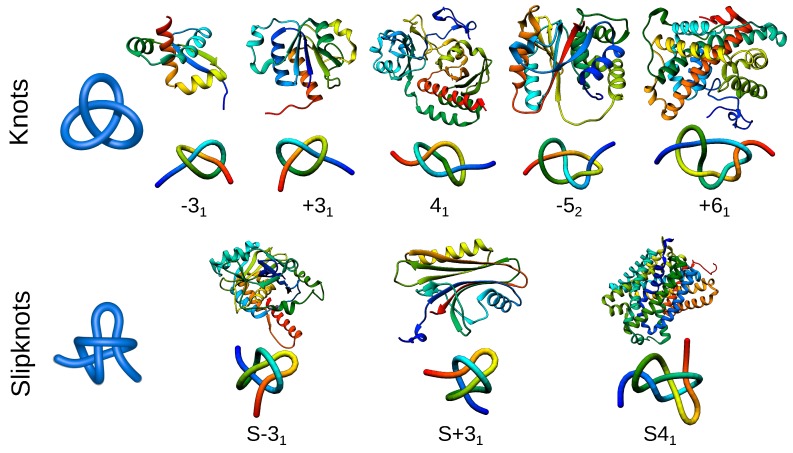
Different types of knotted topologies identified in proteins to date. For each knot/slipknot, its type is indicated by an exemplary structure along with a scheme of its topology. The letter S indicates that the entire polypeptide chain of a given protein forms a slipknot. The type of knot and its chirality (positive or negative) are shown below the schematic figures. PDB IDs of knotted structures from left to right: 2efv, 1j85, 4y3i, 3irt, 3bjx; for slipknotted structures (left to right): 1hyn, 2qqd, 5j4i.

As stated in the Introduction, four types of entanglement in proteins have been identified so far: knots, slipknots, links and lassos, which are presented schematically in Figure 1. They can be found in all kingdoms of life and fulfill a variety of functions, although some trends may be found. For example, over 80% of knotted proteins are enzymes [14] (while this fraction in all PDB-deposited structures is equal to 64%). Knotted topologies have been discussed in a few review articles (e.g., [20,21,22,23,24,25,26,27]), with probably the most up-to-date set of representative, knotted protein topologies presented in [28]. However, the increase in the number of known protein structures forces researchers to constantly search through the PDB and to update the statistics. This is not an easy task since it is very difficult to detect entanglement via visual inspection. To facilitate and automatize the search, various servers and databases have been created, including Knots [29], pKnot [30,31] or KnotProt [14]. The KnotProt is a self-updating database, which also presents biological, structural and sequential information about deposited proteins. Moreover, it also enables studying so-called slipknotted configurations, distinguishable with a (described later in greater detail) knot fingerprint matrix presentation [13,15] (Figure 2).

Proteins with knots and slipknots are classified according to a complexity of topology and geometry, using tools from knot theory. To date, proteins with positive ($+3_{1}$) [11,12,32] or negative ($-3_{1}$) [9,33] trefoil, figure-eight ($4_{1}$) [9], three-twist ($-5_{2}$) [20] or Stevedore’s knots ($-6_{1}$) [33] have been identified (Figure 2). All known slipknotted structures [13,15] feature a $3_{1}$ or a $4_{1}$ knot formed by a sub-chain of a protein. Interestingly, all knots present in proteins are twist knots, and the complexity of the topology depends on the number of twistings of the loop (1/2 for trefoil, full twist for figure-eight knot, etc.). The twist knots have an unknotting number equal to one, which suggests the folding/unfolding pathway, requiring only one threading to tie the protein. The requirement of such a pathway can also explain, why no protein with a non-twist $5_{1}$, $6_{2}$ or $6_{3}$ knot has been discovered, despite the relatively low complexity of such knots. The “twist knots pathway” also suggests that the next identified knots (if any exist) should be one of $7_{2}$, $8_{1}$, $9_{2}$, etc.

**Figure 3:**
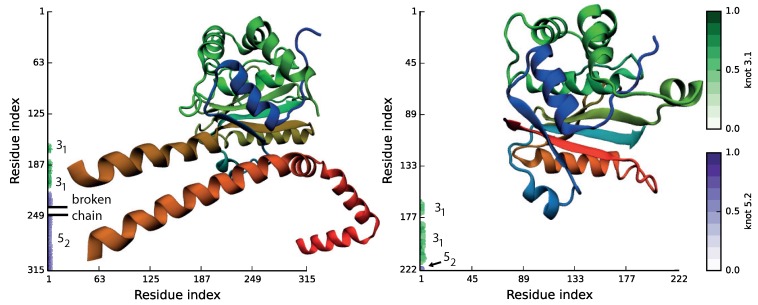
Molecular structures and matrix representation for ubiquitin C-terminal hydrolases from human (PDB ID 4uf5, left) and *Plasmodium falciparum* (PDB ID 2wdt, right). The matrix shows that even though the sequence similarity between proteins is very low, they possess the same knotting motif, K$5_{2}3_{1}3_{1}$.

It is worth noting, that in the strict, topological sense, all (not cyclic) proteins have the same, trivial topology. Therefore, the knotted topology of a protein may be identified only upon appropriate chain closure. This is, however, a non-obvious task, and various algorithms defining knots on open chains have been created, including a direct connection, chain reduction [20], stochastic method [15,34,35], pulling the knot tails [36] and minimally-interfering closure [37]. The topology of proteins can be also described in a graphical way using (mentioned previously) knot fingerprint matrices [13,15], disc matrices [38,39] or using more general mathematical constructs, such as virtual knots [40] or knotoids [41]. The knot fingerprint matrix represents knotting of all sub-chains of a given protein chain. This method is especially useful in the detection of internal knots (slipknots) and in the identification of the same pattern of entanglement. For example, the whole chain in the protein with PDB ID 4uf5 forms a $5_{2}$ knot. Embedded in the structure are, however, two additional trefoil knots (denoted by green color in Figure 3), which can be identified if certain sub-chains are considered. These chains are called internal slipknots. Since the knot fingerprint matrix is constructed by analyzing the topology of every sub-chain, it enables identifying the smallest knotted piece of the chain (knotted core), the knot tails and slipknotted loops. Moreover, representation of a protein via the fingerprint matrix enables comparing the entire topological complexity of a given structure, which can reveal much information about a protein’s function and origin [15]. For example, fingerprints shown in Figure 3 indicate that some proteins with very low sequence similarity could have the same topological complexity.

**Figure 4:**
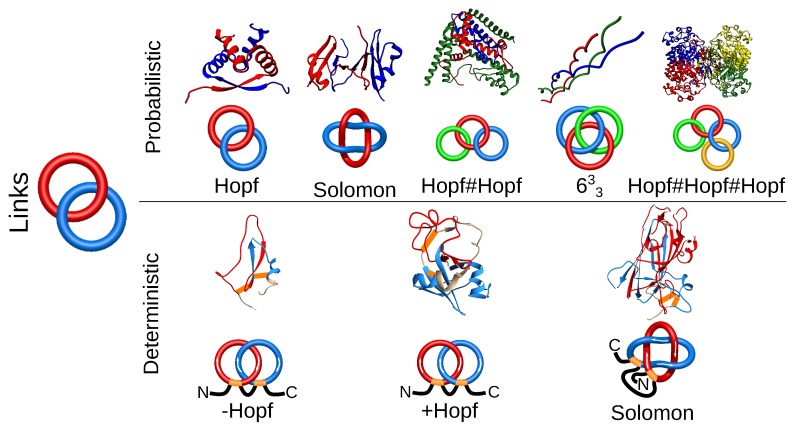
Different types of linked topologies identified in proteins to date. In the top row, the probabilistic links formed by several chains are presented. In the bottom row, deterministic links formed by covalent (disulfide) loops in one chain are shown. For each link type, an exemplary structure along with a scheme of its topology is presented. PDB IDs of the structures of probabilistic links (left to right): 1arr, 2hj1, 5kdm, 3ipn, 2a5h; deterministic links (left to right): 2lfk, 2kqa, 4asl.

Proteins with links are the second class of entangled structures discussed in this review. Links are generalizations of knots; they consist of several components (closed loops) and can also be classified using tools from knot theory. In proteins, several components can be formed from several chains (dimer, trimer, etc.) after their termini are connected. The links formed by several chains may be identified using the extension of chain closure methods, used in the case of knots. Hence, so far, the links were identified by extending the termini and connecting them in the “infinity” (on a surface of a much larger sphere). The direction of the extension was either (historically first) stochastic (with many chain closures) [18] or the termini were extended from the center of mass of a protein [42]. The currently identified set of different multi-component arrangements in proteins is available via the LinkProt database [18] and includes Hopf, Solomon and other more complex links. In principle, the existence of such multi-chain links depends on the closure, and one may introduce a probability cut-off of link existence in the case of the stochastic method. Therefore, such links are called probabilistic links (Figure 4).

In the case of two-component links, also another interesting approach, that of the Gaussian linking number, can be used [43]. The Gaussian linking number is an integer characterizing (up to link homotopy) the mutual arrangement of two (closed) loops. However, this quantity can be calculated using an integral for an arbitrary pair of (even not necessarily closed) chains. For open chains, a usually not easy interpretable non-integer value is obtained. Moreover, this method can require further validation, as, e.g., a Whitehead link cannot be distinguished from unlinked using only the Gaussian linking number.

Another class of links that we identified are deterministic links. Their topology is determined uniquely and does not depend on any arbitrary choices, as the deterministic links are formed by mutual threading of covalent loops (closed, e.g., by disulfide bridges). Such links are formed by single or many protein chains with disulfide linkages. In a single chain, covalent loops forming Hopf or Solomon links have been identified so far [17]. There are also examples of two chains linked by their covalent loops, forming a Hopf [44,45,46] or Solomon [18] link (Figure 4). The identification and classification performed by us for the first time revealed very interesting features of such proteins, e.g., the topologically-induced super-stability (for example, proteins that can withstand boiling in 100 ${}^{\circ }$C without loss of function; see more in Section 3.2) [17].

**Figure 5:**
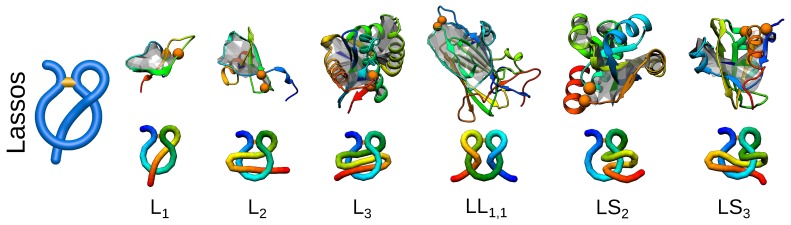
Different types of lassos identified in proteins to date. For each link type, an exemplary structure along with a scheme of its topology is presented. The PDB IDs of the structures (left to right): 1ews, 5uiw, 2ehg, 4a3x, 1zd0, 2msx.

So far, the quest for knots and links was partially suggested by their mathematical classification. However, now, biology seems to outrun mathematics, as the complex lasso structures are biological objects, for which no mathematical classification has been established yet. Complex lasso structures arise in proteins where at least one terminus of a protein chain pierces through a covalent loop closed, e.g., by a disulfide bridge [16,47]. Such a topology has been identified in proteins and classified by us with a computer graphics-derived technique called minimal surface analysis. In this method, a triangulated minimal surface (mimicking a soap bubble) is spanned on the covalent loop, and subsequently, the piercings through such a surface are determined [16]. Using this technique, complex lasso structures may be classified by the number and directions of piercings (threadings) (Figure 5). The current classification of complex lasso structures is available and regularly updated in the LassoProt database [19]. Moreover, the previously mentioned method of the Gaussian linking number can be used for the analysis of complex lasso proteins, where the piercing chain is treated as a part of the loop threading the second, closed, covalent loop. Such an analysis can possibly reveal additional features of the geometry of complex lasso proteins. Furthermore, for some chains, there is more than one pierced lasso loop in one chain. One of the examples is comprised by the deterministic links discussed previously where both loops pierce each other. The possibility of the existence of a few complex lasso loops greatly diversifies the world of complex topology structures, additionally complicating their general classification.

## 3. Advantages and Disadvantages of a Complex Topology

The complex topology of proteins influences their folding, stability and biological properties. The advantages and disadvantages of the complex topology according to recent studies can be divided into four properties: folding, stability, function and conservation. They are summarized in Table 1. Each property influenced by the complex topology is discussed separately in the following sections.

### 3.1. Folding

Folding of proteins can be regarded as diffusion over a free energy landscape [48]. The existence of the topological complexity modulates significantly the shape of the free energy landscape by the addition of the (topological) barriers slowing down the folding. Therefore, it is natural to expect that proteins with a complex topology should require some kind of “help” during folding, e.g., the chaperones. However, it was shown experimentally in vitro that although the addition of chaperones (GroEL-GroES complex) can speed up the folding by an order of magnitude, they are not required for knotted proteins to self-tie [49]. Moreover, in vitro unfolding and successful refolding were performed for a few knotted proteins without any chaperones [49,50,51,52,53,54,55,56,57,58,59,60,61,62,63,64], and in silico folding of knotted proteins was also achieved in a few cases [33,65,66,67,68,69,70,71,72,73,74,75].

**Figure 6:**
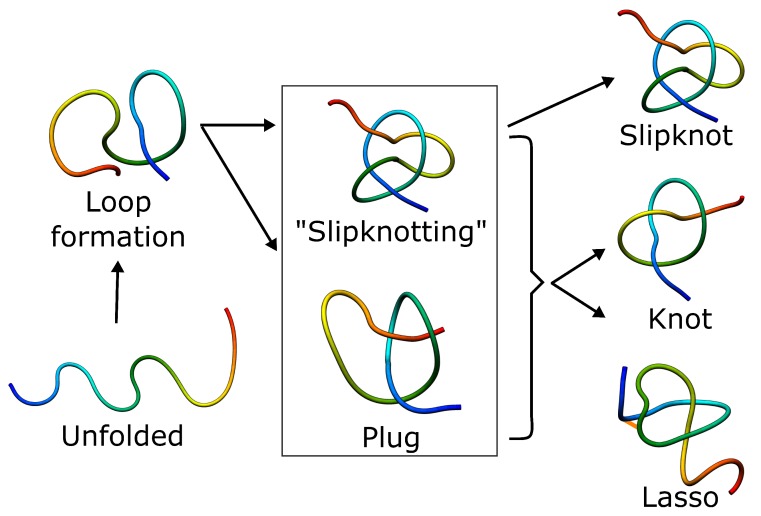
Schematic folding pathways of knots, slipknots and lassos. In each case, folding has to include threading of the chain, which can happen either by slipknot conformation or by direct plugging the chain. The slipknot conformation may then lead to a knot or singly-pierced lasso.

How then can the complex topology protein overcome the free energy barrier and fold effectively? The height of the barrier and roughness on the free energy landscape depend on the topological complexity and the length of the threaded terminus. As threading a longer tail is thermodynamically less favorable, it is usually the shorter terminus that is threaded (according both to theory and experiment). Moreover, the entropic cost to overcome the topological barrier can be further reduced by the adaptation of a hairpin-like configuration, known as a slipknot, while threading (Figure 6) [65]. The “slipknotting” during folding was observed for tails usually longer than 10 residues. Furthermore, knots in proteins arise as a result of a rather precise pathway, contrary to polymers, in which topological complexities are formed based on an ensemble of loose random knots in the first stage, turning into deeper knots after a relatively longer time. This pathway is suggested by the fact that all of the knots represented by proteins are of the twist type [9], and therefore can be tied by the formation of a twisted loop and threading one end through it [65]. The pathway is still accompanied by enthalpic and topological traps, from which proteins can escape using a backtracking mechanism.

The folding, energy landscape and possible traps in the landscape are best understood for small or shallowly-knotted proteins [52,53,54,67,68,71,73,75]. The smallest knotted protein known as MJ0366 [33] serves here as a classic example, based on which it was shown that the protein backbone can self-tie in an explicit solvent molecular dynamics simulation [71]. Self-tying is also well understood for artificially-designed proteins [50,54,55,66,70] and was observed for N-acetylornithine transcarbamylase (AOTCase) [67]. These studies via the comparison to a trivial counterpart have shown that knotted proteins fold more slowly, and their folding is more complicated.

The free energy landscape, however, is less clear for proteins with more complex, but still shallow knots, such as some members of the ubiquitin C-terminal hydrolase family with the $5_{2}$ knot and the protein with the $6_{1}$ knot. In the latter case, computer simulations have shown the possibility of self-tying via a single threading move [33]; however, as suggested experimentally, its folding is accompanied by many intermediates, which can lead to misfolding [76].

Folding of deeply-knotted proteins is still not well understood. In this group, members of the SPOUT methyltransferases family are the most studied, both experimentally [49,56,57,58,59,60] (recently reviewed in [24]) and theoretically [65,74]. The precise self-tying mechanism of deeply-knotted proteins was suggested to follow the slipknotting mechanism; however, its success rate in simulations is low. Enhancement of folding can be however achieved by the addition of some non-native contacts [67,74], and some insights can be gained using lattice simulations [77,78,79]. The rate-limiting step for folding is the knotting, as evidenced by the comparison of the pre-knotted unfolded protein with its artificial, trivial counterpart. In this case both proteins (knotted and unknotted) can smoothly fold to the native conformation [65]. Nevertheless, in vitro experiments have shown that deeply-knotted proteins can fold and self-tie spontaneously, albeit slowly, with significant speed up upon the addition of chaperones [49]. The possibility of self-tying of deeply-knotted proteins with and without chaperones creates the possibility to adjust the folding rate to the needs of the cell. However, no studies have been done so far to show if such a mechanism really exists and is utilized in living cells.

In silico folding of deeply-knotted proteins is a perfect test for folding funnel theory [48] and compatibility with it of the currently existing models. In particular, self-tying of proteins in structure-based models stays in accordance with folding funnel theory showing that native contacts alone are sufficient to tie the protein, and the free energy landscape, shaped by evolution, is minimally frustrated. On the other hand, structure-based models cannot fold deeply-knotted proteins effectively. Moreover, the fraction of native contacts, *Q*, the standard order parameter (grounded in the funnel landscape theory), was shown to be insufficient for a proper description of knotted proteins [72]. This pushes researchers to seek extensions of the native-centric funnel landscape theory by modification of models either by the addition of non-native interactions [67,74] or by selecting only “important” native contacts [72].

Other models concentrate on explaining the role of chaperones on the free energy landscape. According to recent results, the chaperone-induced speed up in kinetics stems probably from the reduced volume of folding, as the simulations show that the confinement [80,81,82] or ribosome-simulating basin [83,84] speeds up the folding of knotted proteins. Moreover, the chaperone confinement can alter the folding pathway. In particular, in the case of the ubiquitin C-terminal hydrolase (UCH) family, some of whose members possess a deep $5_{2}$ knot, the presence of confinement favors the pathway with a $3_{1}$ intermediate product [81], which is substantially less populated in the bulk conditions. The existence of two parallel pathways for UCHs, both with intermediate products, was also observed in experiments [61,62,63,64].

Additionally, the newest survey of knotted proteins reveals the existence of membrane and mitochondrial knotted proteins [28]. This raises a new question: How may the proteins self-tie after passing the pore in the mitochondrial membrane? To our knowledge, this phenomenon has not been studied yet; however, there are some works concerning translocating the knotted polymer through nanopores [85,86,87,88]. On the other hand, it was shown that the interface of two phases (water/membrane) can facilitate self-knotting [89,90].

Although characterization of folding and knotting of proteins still remains a challenge for scientists, some insights can be gained by studying other complex topology proteins. Namely, in the case of complex lasso proteins, a similar threading event is required, if only the covalent loop formation precedes folding of the threading tail. The necessary condition is therefore that the protein should fold in oxidative conditions in which bridges may fold co-translationally. For these proteins, if the folding mechanism is robust, the problem of the bridge formed prior to the core folding must have been solved evolutionarily, and therefore, at least some of those proteins have to efficiently deal with threading of the tail. In fact, the analysis of UniProt entries shows that over 80% of lasso proteins are secreted proteins, for which folding occurs in oxidative conditions. As 18% of proteins with disulfide bridges are complex lasso proteins [16], there is a quite high probability that some structures may be used as templates to study threading, which may shed new light on self-knotting of proteins. This may help overcome the impasse in the topic of knotted protein folding. In fact, some work on studying the folding of complex lasso proteins in vitro has already been done [91].

#### Misfolding and Aggregation

Complicated folding pathways observed in the case of entangled proteins should result in a relatively high probability of misfolding and aggregation. Indeed, the $5_{2}$ knotted UCHs were shown to be aggregation-prone in their intermediate states, and the aggregates may induce various neurodegenerative diseases [92]. Moreover, the existence of the incorrect topology in the unfolded basin and at intermediate states, which could be a reason for such misfolding, was observed in single protein simulations [81].

Misfolding due to topological constraints is well reported in the case of leptin, which possesses the lasso motif [93]. It was also observed during folding of the smallest protein with the deterministic link, tick-derived protease inhibitor (TdPI) [94]. This protein was shown to fold in oxidative conditions mostly to a non-native form, which probably is the structure with a trivial topology (despite native arrangements of disulfide bridges) [17]. This shows that the wrong order of disulfide bond formation leads to misfolding, but on the other hand, the correct topology induces additional stability discussed in the next section.

### 3.2. Stability

The reduction of conformational entropy (which hinders folding), results on the other hand in the increase of protein stability. This is especially evident in the case of deterministic links in which all such proteins are super stable, some withstanding boiling in $100\;{}^{\circ }$C without loss of function [17,44]. The same effect is visible in the case of lasso proteins (especially small lasso peptides), for which the tail is mechanically blocked inside the loop [47,95,96] even after cutting the chain into two parts [97]. The topology-induced stabilizing factor can be also identified in the case of probabilistic links [98]. The increased stability may be a reason why over 80% of lasso proteins are secreted outside the cell and some link proteins act even outside of the host organism [99].

The topologically-induced stability was most extensively studied in the case of knotted proteins. Their increased stability was evidenced both in theoretical [66,100], as well as experimental [13,21,50,101] thermal or chemical unfolding. On the other hand, on-lattice results show that knots may influence the kinetic instead of thermodynamic stability of proteins [26]. Interestingly, the thermal or chemical unfolding of knotted proteins results in polymer-like structures, which still remain knotted [58,59,100,102]. Surprising retention and tying of a deep knot along the protein chain were first noticed via mechanical pulling [103]. Since then, the mechanical stability due to topological constraints was detected in many knotted proteins [100,104,105,106,107]. On the other hand, the mechanical resistance of slipknotted proteins strongly depends on the geometry of a slipknot [108,109]. In proteins where the slipknot loop is much longer than the knotted loop, pulling can lead even to a metastable configuration with a tightened slipknot and high mechanical resistance [108]. This phenomenon does not exist for uniformly-elastic polymers and thus additionally distinguishes entangled proteins. Mechanical resistance of lasso or link proteins according to our knowledge has not been studied. However, such proteins should retain a high mechanical resistance since it was predicted that there is an arrangement of disulfide bridges that leads to a so-called cysteine slipknot motif, which retains a force twice stronger than for titin protein (210 pN) [110].

**Figure 7:**
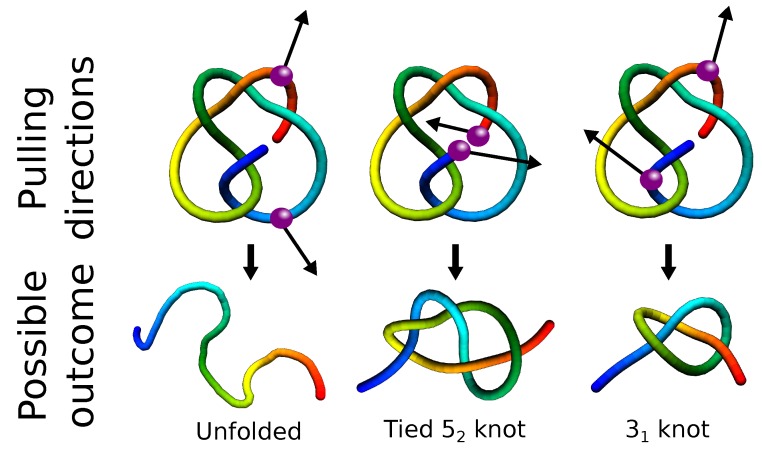
Upper row: Schematic representation of a $5_{2}$ knot identified in UCH proteins, with the spheres and arrows indicating the main geometrical directions of pulling. Bottom row: The resulting final topology, respectively trivial, tied $5_{2}$ and $3_{1}$ knot. Directions of pulling were chosen based on [111].

The investigation of pulling directions may also give a clue of how to solve the problem of the degradation of super-stable knotted proteins. Indeed, increased stability of such proteins and retention of the knot in the unfolded state may hinder degradation, leading to potentially harmful protein accumulation. However, it was shown that pulling in carefully chosen directions may lead to tying or untying of the protein [51,111]. The biological realization of this phenomenon may be the direction of degradation; it was shown that for some proteins, their degradation process depends on the direction of the entry to the proteasome [112,113] (Figure 7). In fact, there were also some simulations concerning the degradation of knotted systems [114]. While this work was being revised, a new article about the degradation of the smallest knotted protein MJ0366 showed that this protein can indeed be degraded only from one terminus [115].

On the other hand, the existence of a knot modulates the effective persistence length of proteins [102]. This introduces a local stiffness of the chain [78], which can also have a functional advantage, especially in the case of proteins subjected to some kind of tensions, e.g., structural proteins.

### 3.3. Function

**Figure 8:**
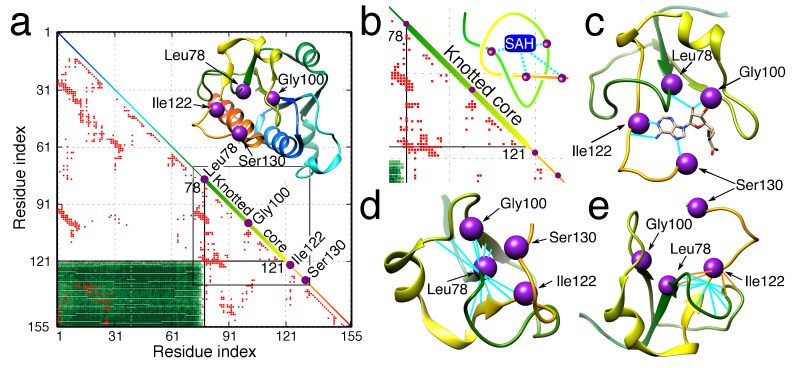
The example of knot-induced advantages for function. (**a**) The knot fingerprint matrix of TrmL methyltransferase with the ligand binding residues denoted by purple dots in the diagonal. The green rectangle denotes the extent of the knotted core. The core is also shown in the diagonal (thick line). The red dots denote the inter-residue contacts. In the inset, the structure of TrmL with the binding residues is marked. The colors in the diagonal correspond to the protein structure. (**b**) The magnification of part of the matrix in (a) (in the square) with the scheme of ligand (SAH) binding. The ligand binding residues are located in or in the vicinity of the knotted core of the protein. (**c**) Structure of the ligand bound to the enzyme. (**d**,**e**) The binding residues located in the termini of the knotted core. The knot enforces a high inter-residue contact density of binding residue and location on the verge of the hydrophobic core of the enzyme, which supports the ligand binding and enzymatic activity of the residues. Figure used with permission from [118].

The forced local rigidity of the chain, in particular, shapes the ligand binding parts of proteins, e.g., active sites of enzymes [11,12,116,117]. In some cases, it is even probable that such an optimal configuration of the chain would not be available without a knot [104,116]. Moreover, the local rigidity of the knot creates places in the structure on the border between the solvent and the hydrophobic protein interior, surrounded by many residues, places characteristic and favorable for enzymatic active sites [118]. This may be the reason why over 80% of proteins (according to the KnotProt) are enzymes (Figure 8), the active sites of which are embedded in the knotted core. It has to be noted that the fraction of enzymes among knotted proteins is much higher than for complex lasso proteins (39%) and among all PDB structures (64%). Not surprisingly, knotted and slipknotted motifs are common in proteins that function in harsh conditions such as low or high pH or a very high temperature.

On the other hand, the global stability of proteins with a complex topology may predestine them to some functions even inside the cell. For example, $5_{2}$ knotted UCH proteins detach the ubiquitin, the degradation marker. Therefore, taking an active part in the degradation process, UCHs probably utilize their increased stability in order not to be degraded themselves. In the case of some proteins that work as transmembrane channels (e.g., protein LeuT(Aa), a bacterial homolog of neurotransmitter transporters), evolution probably used the topological constraints represented by the slipknot loop to strap together the transmembrane helices to form stable functional channels [15]. Another example can be lasso peptides, which, because of their topology, act as molecular plugs for the NTP uptake channel by inhibiting RNA polymerase [119,120,121]. Their efficiency in channel blocking is only due to their topology-induced stability. Analogous activity is characteristic for other lasso proteins with the same $L_{1}$ topology. Similarly, lasso proteins with the $L_{2}$ topology usually are different kinds of signaling proteins [16]. Leptin is one of such proteins, which plays a key role in regulating energy intake/expenditure, metabolism and hypertension [122]. For leptin, it was shown that the lasso topology plays an important role in receptor binding and thus mediates biological activity [91,93]. Another functional advantage of topological constraints is suggestive in the case of proteins with a complex lasso motif, such as supercoiling or double lasso, which cause the adhesion of cells [16]. These proteins are under constant tension, and maybe this is a reason why their structure is additionally stabilized by topological constraints, since not all proteins that interact with a membrane are functional. Moreover, all proteins with the positive Hopf deterministic link, as well as Solomon link proteins are carbohydrate-binding proteins [17]. The origin of this fact is however still unknown.

The function of complex lasso proteins may be also dependent on the conditions, as depending on the oxidizing potential, the crucial lasso-forming bridge may be formed or torn apart, releasing the tail to the solution. This in principle allows for a major allosteric change, which can also be utilized in biotechnology. In particular, a change of topology upon a change of redox potential may be also used to prepare a molecular switch, as suggested in [122]. However, it is probable that the same mechanism is already in use in Nature, but has remained unnoticed due to the fact that complex lassos remained unnoticed until very recently.

### 3.4. Evolutionary Conservation

**Figure 9:**
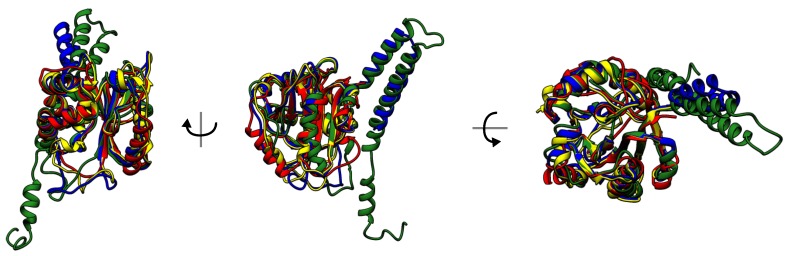
Superimposed structures of UCHs with sequence similarity lower than 30%. Knot-induced rigidity forces the structure conservation.

The fact that all deterministic link proteins with a positive Hopf link bind carbohydrates is one of the examples of exceptional conservation of the structure in complex topology proteins, despite low sequential similarity [17]. In the case of knotted and slipknotted proteins, evolutional conservation of topological constraints is observed for each type of topology over all members, despite very low sequential similarity (lower than 30%) [15], Figure 9. For example, the same complex topological motif (known as $K5_{2}3_{1}3_{1}$) is observed in ubiquitin C-terminal hydrolases from such evolutionarily distant organisms as yeast, *Plasmodium* and human, even though human and yeast proteins have 32% sequence identity, yeast and *Plasmodium* 28% and *Plasmodium* and human (UCH L5) 25%. Another complex topological motif consisting of coupled figure-eight and trefoil slipknots, known as $S3_{1}4_{1}3_{1}$, is strictly conserved across different families of transmembrane proteins and across widely divergent microbes. All of these structures are membrane co-transport proteins.

Evolutionary conservation of a topological motif was also a motivation to extract the SPOUT fold from the Rossmann fold, in the type of AdoMet-dependent MTase structure proteins [123]. The SPOUT methyltransferases (MTases) are a large class of *S*-adenosyl-L-methionine-dependent enzymes that form an alpha/beta fold with a deep trefoil knot embedded in the active site. In the Rossmann fold, the knot is absent. This suggests that structural constraints following from topological complexity allow for higher tolerance of point mutations, as even a high number of them may lead to the same structure of a functional protein. Such mechanism can be one of the ways of indirect protection of crucial enzymes against random mutations, especially in organisms with relatively frequent point mutations.

Another possible advantage of strict conservation of a topological motif relative to sequence is that it allows the optimization of the structure by evolution. This is well seen in the case of slipknotted proteins, where amino acids crucial for function are well conserved on the border of the knotted loop and slipknot loop, while both loops can adopt different sizes and conformations in different species, e.g., members of the sulfatase family. However, someone could see this as a disadvantage since there is only limited space for structure optimization. Nevertheless, the complex topology proteins seems to be well optimized towards the function they fulfill and towards the ligands they bind [116]; therefore, maybe there is no room for improvement already.

On the other hand, strong conservation of structure despite even a large number of single point mutations results in a low number of close homologs with different topologies. In fact, usually the homolog either has the same topology or is totally different structurally. In the case of knotted proteins, there is only one known natural pair of homologous proteins, one being knotted, the other unknotted (acetylornithine transcarbamylase, knotted; and ornithine transcarbamylase, unknotted) [20,100,124] and one artificially-designed knotted protein [50]. This gives researchers a limited possibility to investigate the influence of the topology. Again, however, the lasso proteins may be a remedy, as their topology depends on the oxidizing potential of the solution.

## 4. Discussion

The existence of complex topology proteins remains a challenge for researchers. Only when the scientific community seemed to agree on most aspects of knotted proteins, entirely new complex structures (links and lassos) emerged. In this review, we presented the advantages and disadvantages of a complex topology, starting from folding, to stability, function and evolutionary conservation. The complex topology proteins have many drawbacks; however, in most cases, they can be overcome by some cellular machinery. In particular, although the complex topology proteins may fold slowly, jeopardizing the cell with the high possibility of misfolding and aggregation, utilization of chaperones may substantially facilitate folding. Although complex topology proteins’ degradation may be cumbersome, this problem may be solved by appropriate direction of a protein to enter the proteasome. These issues are not well studied yet; nevertheless, the knot, lasso or link topology in proteins seems not to be really a burden for the cell, and due to many advantages of complex topology proteins, they are more likely an evolutionary achievement, than an artifact predestined for elimination.

From this viewpoint, one may ask: Why are there less knotted proteins than in the case of polymers with similar lengths and properties (such as self-attraction) [125,126,127]? There may be a few answers. First of all, one has to be careful when comparing proteins to polymers. As stated by Flory, there is no other polymer that could adopt a globular configuration stabilized by self-interaction, at the same time transforming reversibly to the random coil [128]. In particular, no standard polymer theory explains why proteins are built of only helices and sheets [129] and what is the possible universe of protein folds [130]. Therefore, the statistics based on polymers may not be accurate for proteins. The use of a long polypeptide with a definite structure (like poly-proline) does not help much, as such constructs are rare or even unnatural and cannot serve as a proper statistics for true proteins with over 20 diversified building blocks (amino acids). Hence, one should analyze the fraction of knotted structures in a large set of random protein sequences. However, a completely random protein sequence in most cases does not fold to some determined structure. Moreover, we do not know, in general, which sequence would fold and which would aggregate, and the proteins we analyze today represent some sort of exceptions, selected, shaped and optimized by evolution during billions of years. Furthermore, the proteins are shaped by evolutionary pressure to be functional. The non-functional proteins, even if they can fold to well-defined structures, are being suppressed. Therefore, the true set to compare should be the set of all evolutionarily possible sequences, folding to determined and functional structures (possibly with the aid of chaperones, which additionally complicates the picture). Such a set is currently impossible to predict.

Secondly, most probably many knotted protein structures remain to be discovered, especially among large proteins, which have not been studied yet due to technical limitations. The recent discovery of three-domain knotted protein [28] shows that many large-sized knotted configurations may await. Therefore, there may be many more knotted, slipknotted, lasso or link proteins than we currently know (which also disallows calculating the proper fraction of complex topology proteins).

Finally, the statistics works for equilibrated sets. However, proteins are still evolving. We cannot tell if all possible complex topology proteins have evolved so far, especially as, due to the conservation of structure upon point mutation, only some rare, serious mutation (e.g., gene duplication) could possibly lead to a new complex topology protein. Therefore, possibly many more knotted proteins will emerge in the next billions of years.

Therefore, possibly, the number of knots in proteins is equal to that formed “randomly” or even knots are suppressed in biology. Possibly, but through the whole review, we tried to convince the reader, the knots and other topological complexity may equip the protein with special features perfectly suited for some particular cases. Additionally, understanding of the topological influence is then crucial for understanding the biology and biophysics of this 6% of proteins, especially as those proteins are promising tools in medicine and materials science. Some entangled proteins whose active site is knotted (e.g., TrmD [116]) are leading antimicrobial drug targets [131]. Moreover, other entangled proteins come from some Gram-negative bacteria, which are responsible for multi-drug resistance, so they are a therapeutic target of high importance, as new drugs are urgently needed, as pointed out by Gretchen Vogel [132]. In materials science, the complex topology structures seem to be a starting point in creating new functional or super-stable materials, and the topology itself may be used to additionally stabilize the existing biopolymers [133]. This may be also useful to prepare super-stable protein-based therapeutics, which could lead to a decrease of the dose needed by patients.

Taking these possible applications into account, we hope and predict that complex topology proteins will become a new, dynamically-expanding branch of science. The validation of the topology of a structure of any polymer may be performed using existing on-line tools implemented within the KnotProt, LassoProt and LinkProt databases. To identify the best place to introduce the mutation for the lasso topology, one may use the PyLasso plugin for PyMOL allowing for easy visualization of lasso structures in any biopolymer [134]. Finally, as the correct topology is crucial for understanding the biology and biophysical properties of proteins, one may repair broken protein chains using the GapRepairer server, which takes into account the topology of the repaired structure [135].

As stated above, there is no perfect polymer analog of proteins. Nevertheless, the results on complex topology polymers or DNA may be a good guidance for what to look for in the world of proteins. From that viewpoint, every result concerning knotted or lasso polymer equilibrium properties [88,136,137,138,139,140], dynamics [141,142], knots in confined space [143,144] or knot interaction [145] may be useful for scientists working in the field of complex topology proteins.

Complex topology proteins are ideal tools to verify the existing models and theories of protein folding and interactions. However, due to the evolutionary conservation (following from topology), there is only a limited number of topologically-trivial homologs, disallowing direct analysis of the influence of the topology. Moreover, the self-tying event remains unspotted in experiments, as currently, the topology may be determined only after folding (usually by crystallization of the protein). Despite assignment of many NMR signals [146] or the preparation of tryptophan variants [147], self-tying still remains elusive, probably due to the complex folding route attained by knotted proteins. It seems like the complex topology, on the one hand, inspires researchers; however, on the other, it prevents them from its study. The complex topology proteins jealously guard their secrets.

## Figures and Tables

**Table 1:** Advantages and disadvantages of the complex topology in proteins.

Property	Advantages	Disadvantages
**Folding**	Possibility to adjust folding time by the change of conditions Pre-knotted conformation serves as a nucleation site for protein folding	Slow folding Higher possibility of misfolding or aggregation Unclear self-tying mechanism May require additional machinery to fold
**Stability**	Resistance to harsh conditions (temperature, pH) Increased mechanical stability Local stiffness	Possible problems with degradation Possible problems with translocation through pores
**Function**	Knot fixes the conformation suitable for ligand binding Knot forms regions favorable for active sites Possibility to switch function (in the case of lasso proteins)	
**Conservation**	High structural conservation despite low sequential similarity High possibility of evolutional optimization of the sequence	Smaller possibility of evolutional optimization of the protein fold
